# Phosphorylation of EF-P aggravates *Streptococcus suis*-induced blood–brain barrier damage by enhancing serine protease production

**DOI:** 10.1186/s13567-025-01612-x

**Published:** 2025-11-07

**Authors:** Hang Yin, Yutong Tian, Zeyu Zhou, Shiqi Lang, Yuqing Li, Lianci Peng, Juan Li, Rendong Fang

**Affiliations:** 1 Joint International Research Laboratory of Animal Health and Animal Food Safety, College of Veterinary Medicine, Southwest University, Chongqing, 400715 China; 2 National Center of Technology Innovation for Pigs, Chongqing, 402460 China

**Keywords:** *Streptococcus suis*, EF-P phosphorylation, serine/threonine kinases, serine protease, blood–brain barrier (BBB)

## Abstract

*Streptococcus suis* (*S. suis*) causes meningitis in humans and pigs. Elongation factor P (EF-P) is an important translation factor that facilitates protein synthesis with polyproline motifs. Posttranslational modification of EF-P is required for its activity and bacterial virulence. However, the regulatory mechanism of EF-P phosphorylation in *S. suis*-induced blood–brain barrier (BBB) disruption remains unclear. Our phosphoproteomic analysis revealed that EF-P was the key substrate of serine/threonine protein kinase (STK) in the SC19 strain. In vitro and in vivo phosphorylation assays revealed that EF-P was phosphorylated by STK at Ser-148 and Thr-176. Compared with the wild-type SC19 strain, the *efp* overexpression mutant strain SC19-(pSET2-EF-P) increased ZO-1 degradation in human brain endothelial cells (hCMEC/D3), the bacterial load, and the mouse survival rate; moreover, blue diffusion in the mouse brain; and brain damage, whereas the *efp* point mutant strain SC19-(pSET2-EF-P-T176A) abolished this enhancement effect. Furthermore, we determined that EF-P regulated the expression of the B9H01_03990 protein, which is defined as a serine protease (SP). Recombinant SP induced ZO-1 degradation in hCMEC/D3. In addition, the *B9H01_03990*-deficient strain (Δ*B9H01_03990*) alleviated ZO-1 degradation in hCMEC/D3 cells and BBB disruption in the mouse brain and transwell infection models, whereas the complementary strain CΔ*B9H01_03990* reversed this reduction in BBB disruption. Our study reveals that *S. suis*-induced BBB disruption is mediated through a novel mechanism involving EF-P phosphorylation and the proposed STK/EF-P/SP signalling axis, providing new insight into the pathogenesis of *S. suis* meningitis.

**Supplementary Information:**

The online version contains supplementary material available at 10.1186/s13567-025-01612-x.

## Introduction

*Streptococcus suis* is an important zoonotic pathogen that causes meningitis in humans and pigs [1]. Disruption of the blood–brain barrier (BBB) is a critical step in meningitis development [2]. The identification of virulence factors associated with BBB disruption has contributed to the development of anti-virulence strategies. Bacterial elongation factor P (EF-P) was first identified in *Escherichia coli* in the 1970s and shares homology with eukaryotic initiation factor 5A (eIF5A) [3], which enhances the translation of polyproline-containing proteins [4]. In the process of synthesizing proteins containing consecutive proline residues in bacteria, ribosomes are stalled, and EF-P is required to continue translation [5]. Bacterial EF-P has been reported to play critical roles in growth, metabolism, stress resistance and pathogenicity [6]. In addition, posttranslational modifications of EF-P are essential for rescuing ribosome stalling [7]. To date, three different types of posttranslational modifications of EF-P in different bacteria have been identified: β-lysylation at lysine 34 in *E. coli*, rhamnosylation at arginine 32 in *Staphylococcus aureus* and 5-aminovalerylation at lysine 32 in *Bacillus subtilis* [8]. However, the effect of the posttranslational modification of EF-P in *S. suis* on its rescue activity remains unknown.

Reversible protein phosphorylation is a pivotal posttranslational modification that controls multiple physiological processes in bacteria, such as cell wall synthesis, oxidative stress, metabolism and virulence [9–11]. *S. suis* expresses phosphosignaling molecules, including serine/threonine kinase (STK) and tyrosine kinase. The potential. Potential substrates of STK in *S. suis*, such as phosphoglucosamine mutase (GlmM) and the GntR transcription factor [12, 13], which are phosphorylated by STK and control *S. suis* pathogenicity, have been identified. However, the regulatory effect of STK on EF-P remains unknown.

In this study, we determined that EF-P is a potential substrate of STK and is phosphorylated at the Thr176 site. EF-P phosphorylation enhances the synthesis of serine protease (SP) in *S. suis*, resulting in disruption of the BBB. Our study reveals the function of EF-P phosphorylation in regulating *S. suis* pathogenesis.

## Materials and methods

### Ethics statements

Female wild-type (WT) C57BL/6 mice were purchased from Chongqing Enswell Biotechnology Co., Ltd. All the mice were maintained under specific pathogen-free (SPF) conditions before being used at 8–10 weeks of age. All the animal experiments were approved by the Southwest University Ethics Committee, Chongqing, China (IACUC-20231215–02).

### Plasmid construction

The plasmids pSET4s-B9H01_03990*,* pSET2-B9H01_03990 and pSET2-EF-P were used for *S. suis* SC19 mutant strain construction, resulting in Δ*B9H01_03990*, CΔ*B9H01_03990* and SC19-(pSET2-EF-P), respectively. The upstream and downstream regions of B9H01_03990 were amplified via PCR from *S. suis* SC19 via PrimeSTAR HS (Takara, Kyoto, Japan) and then fused using the ClonExpress Ultra One Step Cloning Kit (Vazyme, Nanjing, China).

The *efp* and *B9H01_03990* ORFs amplified from the SC19 genome were subsequently cloned and inserted into the expression vectors pET28a and pGEX-4 T-1, respectively, which express His or GST, respectively, and were subsequently used for polyclonal antibody production. All the constructs were confirmed by DNA sequencing. In addition. The primers used in the amplification of target DNA fragments in the present study are listed in Additional file 2.

The mutant plasmids, including EF-P-T12A, EF-P-S26A, EF-P-T35A, EF-P-T144A, EF-P-S148A, EF-P-T176A, EF-P-T180A, and EF-P-S183A, were obtained from BGI (Beijing, China).

### Bacterial strains

The *S. suis* epidemic strain SC19 [14], which has high pathogenicity in humans, mice and pigs, was used in the present study. SC19 and the mutants were cultured in Todd-Hewitt broth (THB) or THA supplemented with 10% fetal bovine serum (FBS) at 37 °C. *E. coli* was cultured in lysogeny broth (LB) or LB supplemented with 1.5% agar. For plasmid maintenance, kanamycin (50 μg/mL) was added to the media of *E. coli* harboring pET28a, and spectinomycin (100 μg/mL) was added to *S. suis* harboring pSET2.

### Phosphoproteomic analysis

*S. suis* SC19 and Δ*stk* in the mid-logarithmic growth phase were incubated in 500 mL of THB medium with vigorous shaking at 37 °C until an optical density (OD) of 0.5 at 600 nm was reached. The bacteria were subsequently harvested by centrifugation at 5000 × *g* for 15 min. After the supernatant was discarded, SDS lysis buffer was added to the bacterial pellet. Next, the samples were homogenized and boiled for 3 min, followed by sonication for 2 min and centrifugation at 16 000 × *g* for 20 min at 4 °C. Finally, the supernatant was collected, and the protein concentration was determined with a BCA protein assay kit. The samples were analysed by capillary liquid chromatography-nanoelectrospray tandem mass spectrometry (nanoLC-MS/MS) using an Orbitrap Astral (Thermo Fisher Scientific, Waltham, MA, USA) coupled with a Vanquish Neo UHPLC (Thermo Fisher Scientific, Waltham, MA, USA) for 240 min.

Phosphopeptides were enriched using the Fe-NTA Phosphopeptide Enrichment Kit (Thermo, USA) according to the manufacturer’s protocol. The eluted phosphopeptides were concentrated under vacuum and dissolved in 10 µL of 0.1% formic acid (FA) for mass spectrometry (MS) analysis. Finally, the MS data were analysed using Spectronaut software.

### *S. suis* mutant strain construction

The Δ*B9H01_03990* strain was constructed using the thermal suicide vector pSET4s. The serine protease gene ID of the sequence from the WT strain SC19 is *B9H01_03990*. The upstream and downstream regions of the *B9H01_03990* gene were amplified using the B9H01_03990-1-F/B9H01_03990-1-R and B9H01_03990-2-F/B9H01_03990-2-R primers (Additional file 2). The PCR products were subsequently cloned and inserted into pSET4s to obtain the *B9H01_03990* knockout vector pSET4S-B9H01_03990. *S. suis* competent cells were prepared and electroporated with pSET4S-B9H01_03990 by a Bio-Rad Gene Pulser Xcell system in THB culture medium in 0.1 cm cuvettes (2 kV, 1000 Ω and 25 μF) [15, 16]*.* The *B9H01_03990* deletion mutant was designated Δ*B9H01_03990*. A complemented strain was generated by cloning the *B9H01_03990* gene using B9H01_03990 3-F/3-R (Additional file 2). The resulting plasmid (pSET2-B9H01_03990) was transformed into the Δ*B9H01_03990* strain to produce the complementary strain CΔ*B9H01_03990*. Δ*B9H01_03990* and CΔ*B9H01_03990* were confirmed by PCR and sequencing.

In addition, the constructed plasmids SC19-(pSET2-EF-P) and SC19-(pSET2-EF-P-T176A) were electroporated into SC19 competent cells and cultured at 37 °C for 12 h. Electroporation was conducted in THB culture medium by a Bio-Rad Gene Pulser Xcell system with 0.1 cm cuvettes (2 kV, 1000 Ω and 25 μF). These constructed mutant strains were stored in our laboratory.

### Bacterial growth curve

Bacterial suspensions of SC19 (wild-type), SC19-(pSET2-EF-P) and SC19-(pSET2-EF-P-T176A) were incubated in THB supplemented with 10% FBS at 37 °C with continuous shaking for 0, 1, 2, 3, 4, 6, and 8 h. After incubation. The absorbance of the bacterial suspension at 600 nm was measured using a spectrophotometer.

### Protein expression and purification

The primers used in the amplification of target DNA fragments in the present study are listed in Additional file 2. The coding sequences of *stk* and *efp*, *B9H01_03990,* were amplified from the SC19 genome via the primers nSTK-F/nSTK-R and EF-P-F/EF-P-R, B9H01_03990-F/B9H01_03990-R, respectively. The PCR products of nSTK, EF-P, and B9H01_03990 were then each inserted into the digested pET28a or pGEX-4 T vector.

To purify GST-nSTK, GST-EF-P and His-B9H01_03990 cultures of BL21 (pGEX-nSTK, pGEX-EF-P and pET28a-B9H01_03990) were grown to an OD_600_ of 0.8 in Luria broth (LB) at 37 °C with shaking at 180 rpm and then induced with IPTG at a final concentration of 1 mM at 37 °C and 180 rpm for 4 h. Protein purification was performed using Ni Elite Beads (Vazyme, Nanjing, China) and BeyoGold™ GST-tag Purification Resin (Beyotime, China).

### Polyclonal antibody preparation

The pGEX-4 T-EF-P and pET28a-His-B9H01_03990 plasmids were constructed by homologous recombination and transformed into *E. coli* BL21(DE3). The GST-tagged proteins were purified using GST affinity resin (Beyotime, Shanghai, China), whereas the His-tagged proteins were purified using Ni–NTA beads (Ni Elite Beads, Vazyme, Nanjing, China). C57BL/6 mice were subcutaneously immunized with 100 μg of purified EF-P or SP emulsified in adjuvant three times at 0, 14 and 28 days. Finally, the serum was collected 7 days after the third immunization.

### In vitro phosphorylation assay

To detect the phosphorylation of STK and EF-P, 50 μg of purified recombinant EF-P protein or 150 μg of purified STK protein were incubated in 50 μL of phosphorylation buffer (100 mM Tris–HCl pH 8.0, 10 mM MgCl₂, 25 mM NaCl, 10 mM ATP and 1 mM DTT) or without phosphorylation buffer for 2 h at 37 °C. Phosphorylation buffer was procured on ice. Then, SDS loading buffer (5 ×) was added, and the samples were boiled at 95 °C for 5 min. To exclude the effects of autophosphorylation of STK and EF-P, only STK or EF-P was added. Next, the samples were separated on 8% Tris–glycine SDS-PAGE gels and 8% Phos-tag^™^ gels containing 100 μM Phos-tag^™^ acrylamide and 200 μM MnCl₂ (APExBIO, USA), which specifically retards the migration of phosphorylated proteins. Finally, the proteins were visualized by Coomassie Brilliant Blue R-250 staining for 1 h, followed by destaining (40% methanol, 10% acetic acid) for 2‒4 h.

### Immunoblot analysis of EF-P phosphorylation in* S. suis*

SC19 and Δ*stk* were grown to the mid-logarithmic phase with an OD of 0.6, and bacteria were harvested by centrifugation at 4000 × *g* for 10 min at 4 °C. The bacteria were subsequently washed twice with ice-cold PBS and resuspended in 1 mL of PBS containing lysozyme (20 mg/mL) and protease inhibitor cocktail (Beyotime, China). Next, the samples were sonicated on ice using a 3-mm probe at 30% amplitude for 10 cycles (10 s pulse, 10 s rest). Lysates were collected by centrifugation at 12 000 × *g* for 15 min at 4 °C. The protein concentration was determined with a BCA protein assay kit. The total protein (1 mg) and anti-EF-P polyclonal antibodies were incubated with Protein A + G agarose (Beyotime, China) overnight at 4 °C. The beads were washed three times with 1 mL of ice-cold RIPA buffer (50 mM Tris–HCl pH 8.0, 150 mM NaCl, 1% NP-40, 0.5% sodium deoxycholate). The bound proteins were eluted by boiling in 2 × Laemmli buffer (65 mM Tris–HCl pH 6.8, 2% SDS, 10% glycerol, and 5% β-mercaptoethanol) at 95 °C for 10 min. Finally, the samples were subjected to immunoblotting analysis using primary Abs, including anti-EF-P (1:1000) and anti-phospho-(Ser/Thr) (Abcam ab17464, 1:2000) and HRP-conjugated secondary antibodies (1:2000, Beyotime, China).

### *S. suis* infection and serine protease (SP) stimulation in cells

Human cerebral microvascular endothelial cells (hCMEC/D3, JNO-H0520, Jennio Biotech) were cultured in DMEM (Gibco, USA) supplemented with 10% FCS and 1% penicillin/streptomycin (Gibco, USA) and maintained in a humidified 37 °C incubator with 5% CO_2_. The cells were washed three times with PBS and then infected with *S. suis* at a multiplicity of infection (MOI) of 10 for 2 h or treated with purified serine protease at 10, 20, 40, 80, or 100 μg/mL for 2 h. After infection or SP treatment, the lysates were collected for assays as described below.

### Western blot

After treatment with *S. suis* or Δ*B9H01_03990*, hCMEC/D3 cells were lysed in 1 × SDS buffer supplemented with protease inhibitors. Then, the samples were subjected to SDS‒PAGE and subsequently transferred onto a polyvinylidene difluoride (PVDF) (0.45 μm pore size, Millipore) membrane by electroblotting. Next, the membranes were blocked with 5% nonfat dry milk and then immunoblotted with the indicated primary antibodies (Abs), including anti-ZO-1, anti-phos Ser/Thr and anti-EF-P, overnight at 4 °C. The next day, the blots were incubated with horseradish peroxidase-conjugated goat anti-mouse/rabbit IgG (1:2000 Beyotime, China). Finally, the distinct protein bands were detected with SuperKine™ West Femto Maximum Sensitivity Substrate (Abbkine, China) and captured with a ChemiDoc Imaging System (Bio-Rad).

### Quantitative real-time polymerase chain reaction (qRT‒PCR)

SC19, SC19-(pSET2-EF-P), and SC19- (pSET2-EF-P-T176A) cells were lysed, and total RNA was extracted with a bacterial RNA kit (Omega, USA) according to the manufacturer’s instructions. cDNA was synthesized by HiScript III RT SuperMix for qPCR (Vazyme, Nanjing, China) and detected by ChamQ SYBR qPCR Master Mix (Vazyme) using the CFX96 (Bio-Rad, USA). The relative gene expression level was normalized against the expression level of 16S RNA.

### Immunofluorescence microscopy

hCMEC/D3 cells were treated with recombinant SP protein at 0, 10, or 20 μg/mL in serum-free Endothelial Cell Medium (ECM) for 2 h and then fixed with 4% paraformaldehyde (PFA) in PBS for 15 min at room temperature (RT). After three washes with PBS, the cells were permeabilized with 0.1% Triton X-100 in PBS for 5 min and blocked with 5% BSA for 1 h. Then, the cells were incubated with a primary rabbit anti-ZO-1 antibody (1:200, Proteintech 21773–1-AP) at 4 °C overnight in 5% BSA, followed by incubation with an Alexa Fluor 488-conjugated goat anti-rabbit IgG (1:500, ABclonal AS111) for 1 h at RT. Finally, the cells were washed and maintained in Antifade Mounting Medium (Beyotime, China). The cells were observed via fluorescence microscopy (Olympus, Tokyo, Japan).

### In vitro endothelial cell permeability assay using a transwell model

hCMEC/D3 cells were seeded at 5 × 10^4^ cells/insert on collagen-coated Transwell-Clear inserts (3.0 μm pore size, Corning, USA) and cultured for 7‒10 days in ECM. Transendothelial electrical resistance (TEER) was monitored daily by a Millicell ERS-2 Voltohmmeter (Millipore), in which TEER values greater than 200 Ω/cm^2^ and variations in TEER values less than 5% over 3 consecutive days were confirmed as mature barriers. Recombinant SP protein at 20 μg/mL or bacteria in serum-free ECM were added to the upper chamber. TEER was measured at 0, 1, 2, 3, and 4 h posttreatment under standardized conditions at 37 °C.

### *S. suis* infection in vivo

WT C57BL/6 mice were intraperitoneally infected with 100 μL of SC19, SC19-(pSET2-EF-P), SC19-(pSET2-EF-P-T176A), Δ*B9H01_03990, or the C*Δ*B9H01_03990* strain (1 × 10^8^ CFU), and PBS was used as a blank control. For the survival rate, the mice (total *N* = 30 in each group, *n* = 10) were monitored daily for 7 days. After 48 h of infection, the mice (total *N* = 90 per group, *n* = 3) were sacrificed by cervical vertebral dislocation, and brain tissues were collected to determine the bacterial load. In addition, brain tissues were fixed in 10% formalin and then processed in paraffin for hematoxylin and eosin (H&E) staining.

To determine the permeability of the BBB in the mouse brain, Evans blue (EB) staining was performed. WT C57BL/6 mice (total *N* = 72 per group, *n* = 3) were injected with 200 μL of 1% Evans blue (Sigma) via the tail vein at 48 h post infection. Two hours after EB administration, the mice were transcardially perfused with sterile PBS and then euthanized. The brains were subsequently removed and imaged using a digital camera.

### Statistical analysis

The data are presented as the mean ± SEM of three independent experiments (in vitro) for each group (*n* = 3). One-way ANOVA was used to analyse the significant differences for comparisons among different groups. Statistical significance is shown as *P ≤ 0.05, ** *P* ≤ 0.01, ***P ≤ 0.001, ns = not significant.

## Results

### Identification of protein phosphorylation regulated by STK

**Figure 1:**
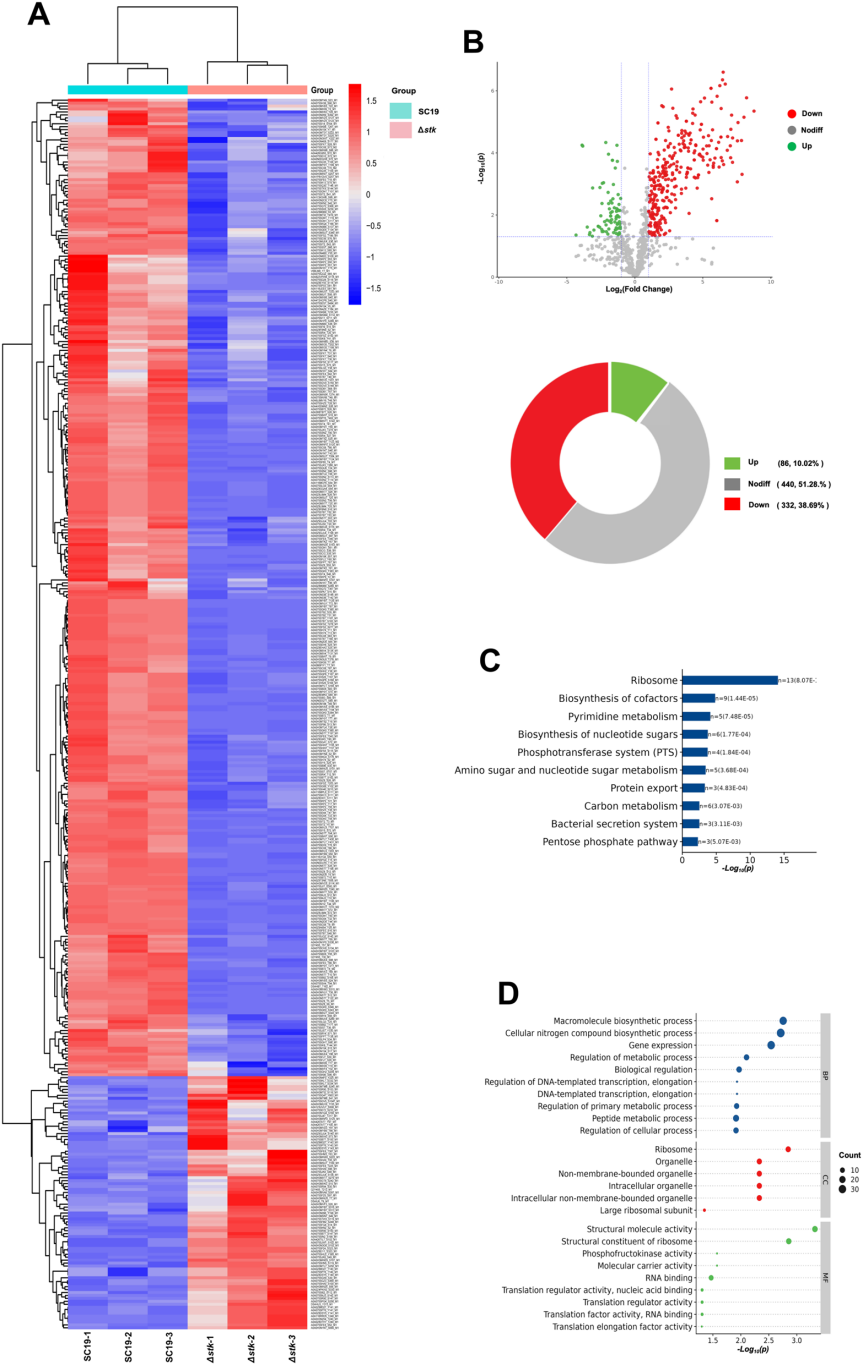
**Phosphoproteomics profiling reveals STK-dependent phosphorylation events**. **A** Heatmap analysis of 418 differentially phosphorylated proteins between SC19 and Δ*stk*. Downregulated phosphoproteins in Δ*stk* are clustered. **B** Volcano plot analysis of differentially phosphorylated proteins (DPPs) including 332 downregulated and 86 upregulated proteins in Δ*stk* compared to SC19. **C** KEGG pathway enrichment analysis of DPPs in Δ*stk* (*P* < 0.05). **D** The top significantly enriched GO terms of DPPs in biological process, cellular component, and molecular function categories (*P* < 0.05). Data are representative of one independent experiment with triplicate samples per group.

To identify potential substrates of STK and its target phosphosites in *S. suis* (SC19), 4D-label free phosphoproteomic analysis was performed in SC19 and Δ*stk*. The results revealed 418 differentially phosphorylated proteins between SC19 and Δ*stk* (Figure 1A). Among these differentially phosphorylated proteins, compared with SC19, Δ*stk* had 332 downregulated phosphorylated proteins and 86 upregulated phosphorylated proteins (Figure 1B). KEGG pathway enrichment analysis revealed that these downregulated phosphorylated proteins were enriched in ribosome, biosynthesis of cofactors, pyrimidine metabolism, biosynthesis of nucleotide sugars, the phosphotransferase system (PTS), amino sugar and nucleotide sugar metabolism, protein export, carbon metabolism, the bacterial secretion system, and the pentose phosphate pathway (Figure 1C). Gene Ontology (GO) enrichment analysis revealed that these downregulated phosphorylated proteins were enriched in terms such as macromolecule biosynthetic process; cellular nitrogen compound biosynthetic process; gene expression; regulation of metabolic process; biological regulation; regulation of DNA-templated transcription; regulation of primary metabolic process; peptide metabolic process; and regulation of cellular process. These results indicate that STK plays an important role in regulating protein synthesis (Figure 1D).

### EF-P is phosphorylated by STK

**Figure 2:**
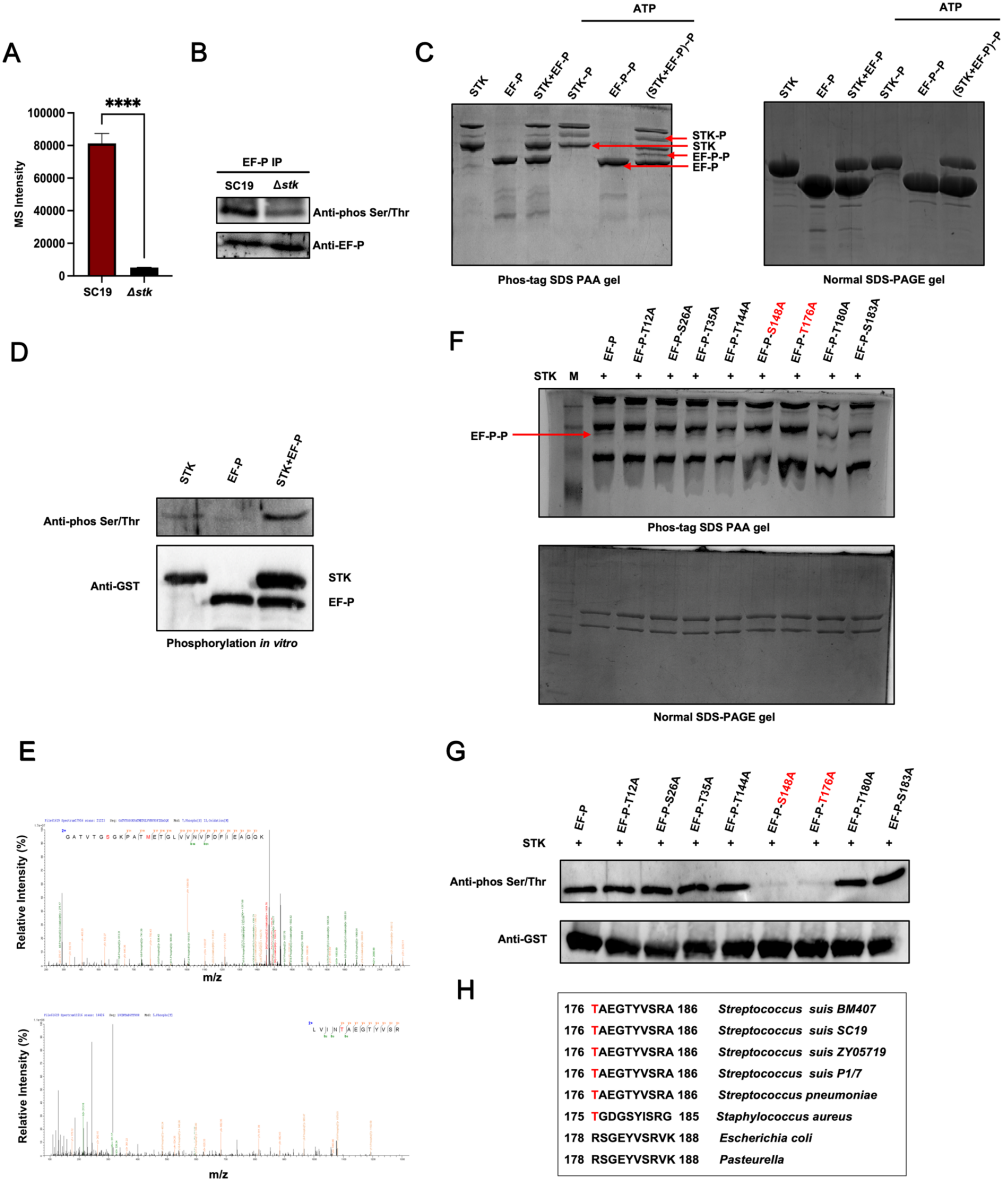
**EF-P is phosphorylated by STK**. **A** Reduced EF-P phosphorylation in Δ*stk* by phosphoproteomics analysis. **B** Coimmunoprecipitation (Co-IP) analysis reveals reduced EF-P phosphorylation in vivo in Δ*stk*. **C** Phos-tag SDS‒PAGE analysis shows direct EF-P phosphorylation by STK. Mobility shift (arrow) indicates EF-P phosphorylation when STK was present. **D** In vitro phosphorylation assay shows direct EF-P phosphorylation by STK. Western blotting shows STK phosphorylates EF-P in kinase reactions. **E** Mass spectra showing that EF-P is phosphorylated at Thr-12, Ser-26, Thr-35, Thr-144, Ser-148, Thr-176, Thr-180 and Ser-183. **F** STK and EF-P, EF-P-T12A, EF-P-S26A, EF-P-T35A, EF-P-T144A, EF-P-T176A, EF-P-S148A, EF-P-T180A or EF-P-S183A were coincubated, separated by Phos-tag SDS-PAA gels, and stained with Coomassie blue. **G** Phosphorylation of EF-P at S148 and T176 induced by STK in vitro. **H** Sequence alignment of the conserved T176 containing region in EF-P orthologues of different species.

Among these potential substrates, protein EF-P (the product of ZY05719_RS06945) was selected for further exploration. The phosphoproteomic results indicated that the phosphorylation level of EF-P was significantly lower in the knockout mutant strain Δ*stk* than in the wild-type strain SC19 (Figure 2A). To determine whether STK affects the phosphorylation level of EF-P in vitro, Co-IP was performed. The results revealed that, compared with that of SC19, the phosphorylation level of EF-P in *Δstk* was lower (Figure 2B). To further determine whether EF-P is phosphorylated by STK, recombinant EF-P was expressed, purified and incubated with STK in vitro. The phosphorylation of EF-P by STK was determined by Phos-tag SDS-PAA gels. Compared with EF-P alone, the phos-tag gel of EF-P with STK presented a migratory band (Figure 2C). In addition, an in vitro phosphorylation assay revealed that STK directly phosphorylated EF-P (Figure 2D).

These results indicate that EF-P is phosphorylated by STK. Next, mass spectrometry analysis was performed to identify the sites of EF-P phosphorylated by STK. The results revealed that Thr-12, Ser-26, Thr-35, Thr-144, Ser-148, Thr-176, Thr-180, and Ser-183 of EF-P are STK phosphorylation sites (Figure 2E). To verify these phosphorylation sites, we constructed site-directed mutants of EF-P in which Thr and Ser were replaced with Ala, including T12A, S26A, T35A, T144A, S148A, T176A, T180A and S183A. These recombinant EF-P mutants were expressed, purified and incubated with STK in vitro, and the phosphorylation of the EF-P mutants by STK was subsequently determined by Phos-tag SDS-PAA gels. Compared with those of WT EF-P with STK, the phosphorylated bands of S148A or T176A with STK on the Phos-tag SDS-PAA gel were diminished (Figure 2F), indicating that S148 and T176 of EF-P are potential STK phosphorylation sites. The phosphorylation of EF-P at S148 and T176 was also confirmed by western blotting (Figure 2G). To assess the conservation of EF-P, we performed multiple sequence alignments of EF-P homologues from different *S. suis* subtypes and other bacteria, including *Streptococcus pneumoniae*, *Staphylococcus aureus*, *E. coli* and *Pasteurella*, which indicated phosphorylation at T176 (Figure 2H). In *S. suis*, the T176 site is conserved across all the examined serotypes, indicating its significant evolutionary conservation. This conservation pattern is also observed in other Gram-positive bacteria, including *Streptococcus pneumoniae* and *Staphylococcus aureus*. In contrast, Gram-negative bacteria such as *E. coli* and *Pasteurella multocida* lack a threonine residue at the corresponding position. This distinct phylogenetic pattern suggests that phosphorylation of T176 constitutes a key conserved regulatory mechanism in Gram-positive bacteria, particularly within the genus *Streptococcus*.

### EF-P phosphorylation enhances *S. suis*-induced blood–brain barrier disruption

**Figure 3:**
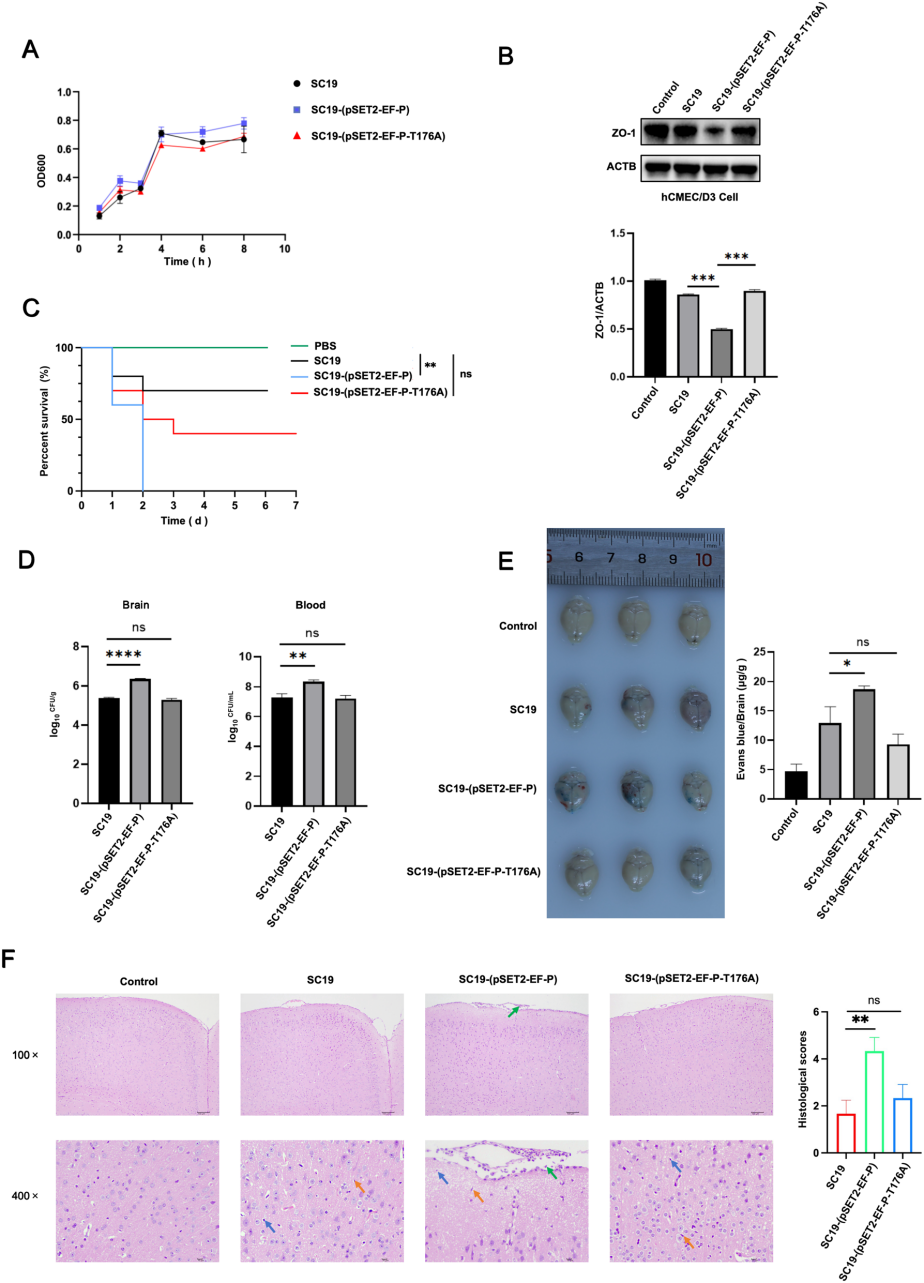
**EF-P phosphorylation enhances** ***S. suis*-induced blood–brain barrier disruption**. **A** Growth kinetics of SC19, SC19-(pSET2-EF-P) and SC19-(pSET2-EF-P-T176A). **B** Western blot showing expression level of ZO-1 in hCMEC/D3 cells infected with SC19, SC19-(pSET2-EF-P) and SC19-(pSET2-EF-P-T176A). **C** Survival curve of mice infected with WT, SC19-(pSET2-EF-P), and SC19-(pSET2-EF-P-T176A). **D** Bacterial load in the blood and brain of mice infected with SC19, SC19-(pSET2-EF-P) and SC19-(pSET2-EF-P-T176A) for 48 h (total *N* = 27, each group *n* = 3). **E** Evans blue (EB) diffusion of mice brain infected with SC19, SC19-(pSET2-EF-P) and SC19-(pSET2-EF-P-T176A) (total *N* = 36, each group *n* = 3). **F** Representative images of H&E staining showing the pathological changes of mice brain tissues (total *N* = 36, each group *n* = 3). **A** and **B** are representative of three independent experiments with triplicate samples per group. **C**–**F** are representative of three independent experiments with three mice or ten mice per group. *P ≤ 0.05, **P ≤ 0.01, ***P ≤ 0.001.

To investigate the effect of phosphorylated EF-P on *S. suis* pathogenesis via disruption of the BBB, we constructed SC-19 mutants with chromosomally integrated pSET2-EF-P or pSET2-EF-P-T176A to mimic phosphorylated and nonphosphorylated EF-P, respectively. First, we assessed the effect of EF-P on bacterial growth. The results revealed that the EF-P mutation did not affect bacterial growth (Figure 3A). However, SC19 slightly downregulated the expression of the tight junction (TJ) protein ZO-1 in human brain endothelial cells (hCMEC/D3), and SC19-(pSET2-EF-P) enhanced the downregulation of ZO-1 expression, whereas SC19-(pSET2-EF-P-T176A) reversed the downregulation of ZO-1 expression (Figure 3B). In addition, SC19-(pSET2-EF-P) reduced the mouse survival rate compared with SC19, whereas SC19-(pSET2-EF-P-T176A) increased the mouse survival rate (Figure 3C), demonstrating that EF-P phosphorylation enhances *S. suis* pathogenicity.

To further assess the effect of EF-P on the integrity of the BBB in vivo, mice were intraperitoneally infected with *S. suis* followed by Evans blue (EB) administration via the tail vein at 48 h post infection. SC19-(pSET2-EF-P) increased the bacterial load in the mouse brain and blood compared with SC19, whereas SC19-(pSET2-EF-P-T176A) abolished this effect (Figure 3D). Similarly, SC19-(pSET2-EF-P) increased EB diffusion in the brain compared with SC19, whereas SC19-(pSET2-EF-P-T176A) reversed this increase (Figure 3E). Furthermore, SC19-pSET2-EF-P induced more severe meningeal destruction, inflammatory cell infiltration (green arrows), necrotic cell debris (blue arrows) and reactive gliosis (yellow arrows) (Figure 3F). These results indicate that the phosphorylation of EF-P at T176 plays an important role in mediating *S. suis*-induced BBB disruption.

### EF-P phosphorylation enhances the expression of serine proteases

**Figure 4:**
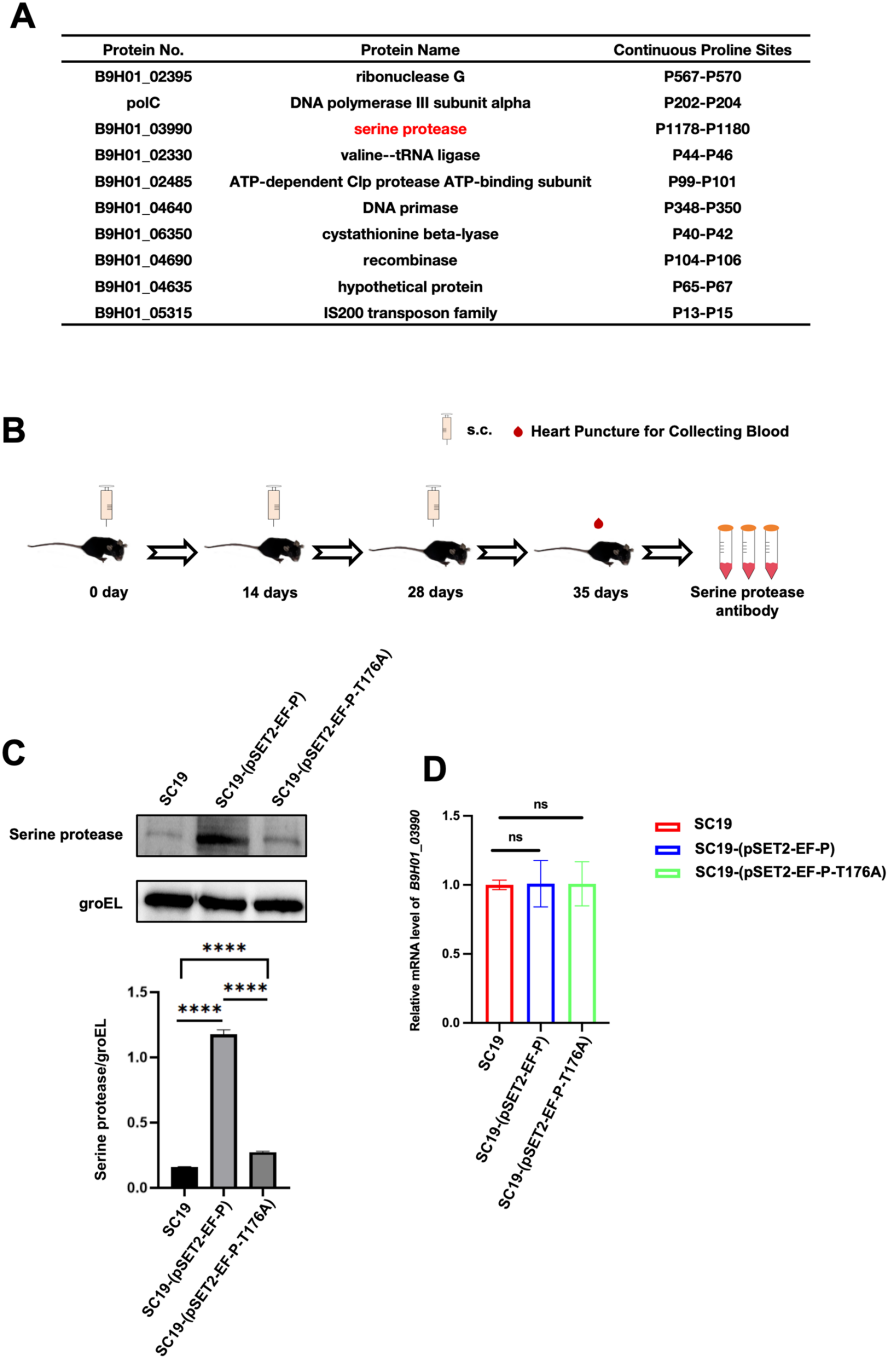
**EF-P phosphorylation enhances the expression of serine protease**.** A** whole-genome sequencing analysis of SC19 shows identified proteins with enriched polyproline motifs. B9H01_03990 (annotated as serine protease, SP) was selected for further investigation. **B** Schematic representation of polyclonal antibody production against SP (B9H01_03990). **C** Immunoblot analysis of SP expression SC19, SC19-(pSET2-EF-P), and SC19-(pSET2-EF-P-T176A). **D** SP mRNA expression in SC19, SC19-(pSET2-EF-P), and SC19-(pSET2-EF-P-T176A). **C** and **D** are representative of three independent experiments with triplicate samples per group. *P ≤ 0.05, **P ≤ 0.01, ***P ≤ 0.001.

Posttranslational modifications of EF-P have been shown to mediate protein synthesis [6]. These modifications enable EF-P to alleviate ribosome stalling by stabilizing ribosome dynamics and promoting peptide bond formation at polyproline motifs, thereby ensuring efficient translation elongation and protein production, indicating a critical role in the synthesis of polyproline-rich proteins [17]. Therefore, we screened proteins B9H0_02395, polC, B9H01_03990, B9H01_02330, B9H01_02485, B9H01_04640, B9H01_06350, BH901_04690, B9H01_04635, and BH901_05315 with enriched polyproline motifs via whole-genome sequencing (WGS) analysis of SC19 (Figure 4A). Serine protease (SP) has been reported to play an important role in *Mycobacterium tuberculosis*-induced destruction of lung epithelial cells. SP cleaves E-cadherin, enhances bacterial colonization and induces lung damage, thereby disrupting the epithelial barrier [18]. Therefore, we selected serine protease (SP) as our protein of interest. The protein B9H01_03990 was annotated as a serine protease on the basis of bioinformatic analyses. Next, we generated a polyclonal antibody against SP (Figure 4B). We found that, compared with SC19, SC19-(pSET2-EF-P) upregulated SP expression, whereas SC19-(pSET2-EF-P-T176A) reversed this effect (Figure 4C), demonstrating that EF-P mediates SP expression. However, EF-P phosphorylation did not affect SP transcription (Figure 4D).

### Serine proteases regulate *S. suis*-induced blood–brain barrier disruption

**Figure 5:**
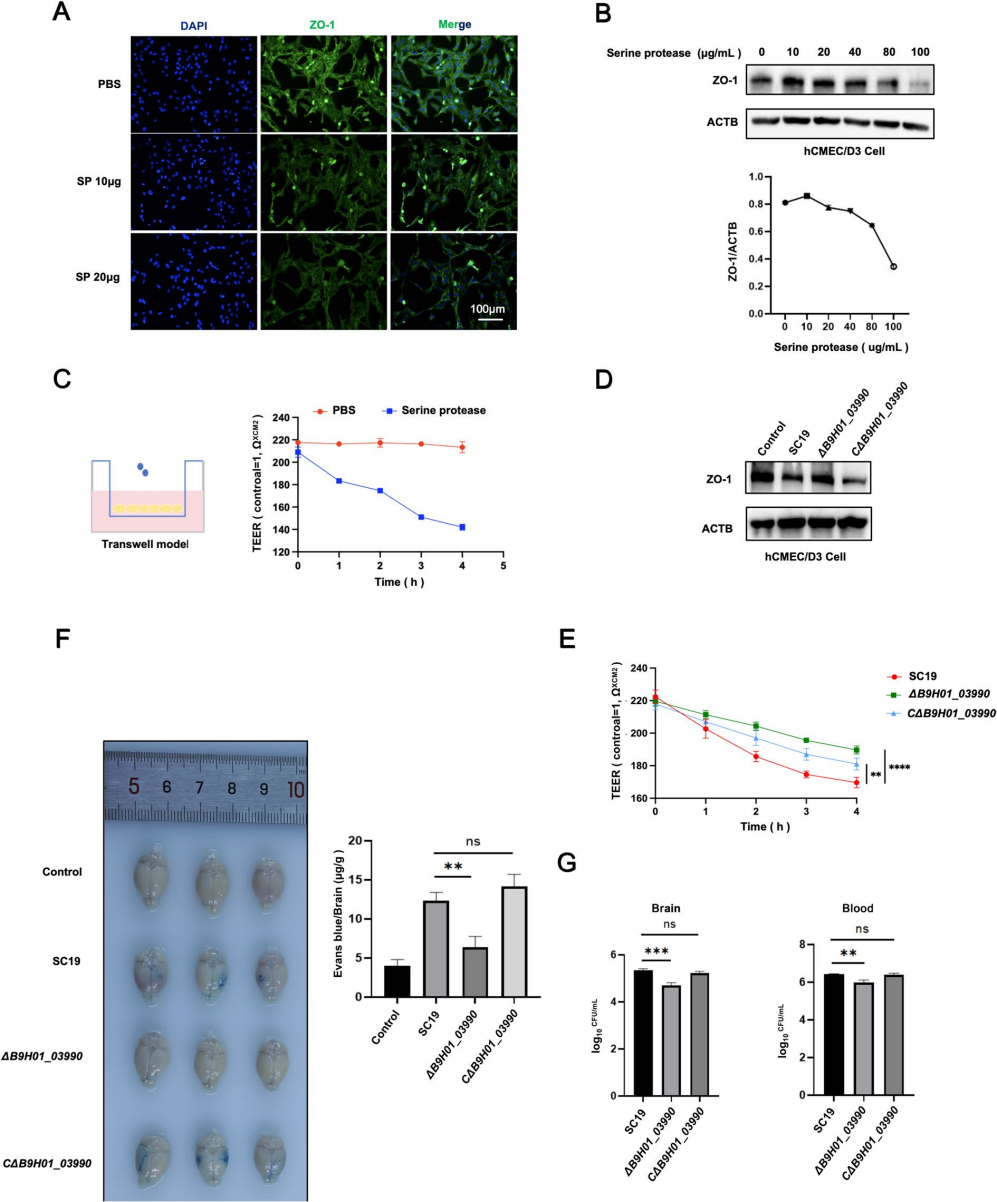
**Serine protease regulates *S. suis*-induced blood–brain barrier disruption**. **A** Representative images of immunofluorescence staining of ZO-1 in hCMEC/D3 cells treated with recombinant serine protease under indicated concentrations. **B** Western blotting showing ZO-1 expression in hCMEC/D3 cells treated with recombinant serine protease under indicated concentrations. **C** The TEER value in the absence and presence of serine protease (40 µg/mL) in transwell model. **D** Western blotting analysis of ZO-1 expression in hCMEC/D3 cells infected with SC19, Δ*B9H01_03990* and CΔ*B9H01_03990*. **E** The TEER value in the transwell model infected with SC19, Δ*B9H01_03990*, and CΔ*B9H01_03990*. **F** Evans blue diffusion of mice brain infected with SC19, Δ*B9H01_03990* and CΔ*B9H01_03990* (total *N* = 36, each group *n* = 3). **G** bacterial load in the blood and brain of mice infected with SC19, Δ*B9H01_03990* and CΔ*B9H01_03990* (total *N* = 27, each group *n* = 3). **A**–**D** are representative of three independent experiments with triplicate samples per group. **F** and **G** are representative of three independent experiments with three mice per group. *P ≤ 0.05, **P ≤ 0.01, ***P ≤ 0.001.

To further assess the impact of the SP protein on the BBB, a transwell model in hCMEC/D3 cells was used to detect the permeability of the BBB using recombinant SP. First, we observed that SP at 10 μg and 20 μg downregulated ZO-1 expression in hCMEC/D3 cells through immunofluorescence microscopy (Figure 5A). Similarly, the results of western blotting revealed that SP downregulated ZO-1 expression in a dose-dependent manner (Figure 5B). In the BBB transwell model, SP significantly reduced the transendothelial electrical resistance (TEER) value in hCMEC/D3 cells (Figure 5C). These results demonstrate that SP may be involved in the destruction of the BBB. In addition, *B9H01_03990* gene deletion upregulated the expression of ZO-1 in hCMEC/D3 cells compared with that in SC19 cells, whereas CΔ*B9H01_03990* reversed this upregulation (Figure 5D). In addition, Δ*B9H01_03990* significantly increased the TEER values compared with those of SC19, whereas CΔ*B9H01_03990* induced a reduction in the TEER values (Figure 5E). Furthermore, compared with SC19, Δ*B9H01_03990* reduced EB diffusion and the bacterial load in the brain, whereas CΔ*B9H01_03990* reversed this reduction (Figures 5F, G). These results indicate that SP is involved in *S. suis*-induced BBB disruption.

## Discussion

EF-P is a highly conserved protein in bacteria and is homologous to elF5A in eukaryotes [19], which prevents ribosome stalling at polyprolines by stabilizing the P-site tRNA and promoting favourable geometry for peptide bond formation. The loss of EF-P results in growth defects or lethal effects on some bacterial species, such as *Acinetobacter baumannii*, *Neisseria meningitidis* and *Mycobacterium tuberculosis*, but does not affect other bacterial species, such as *Salmonella enterica*, *Escherichia coli* and *Bacillus subtilis*. These varied functions are mediated by the modification of EF-P. Although the physiological function of EF-P in bacteria is conserved, the posttranslational modification of EF-P is highly divergent between species. Our study identified a novel posttranslational modification pathway of EF-P in *S. suis* and reported that EF-P phosphorylation mediated *S. suis* pathogenesis.

EF-P modification in different bacterial species is thought to be required for its activity. In *E. coli* and *Salmonella*, EF-P activity is dependent on β-lysylation by the YjeK/YjeA complex, and unmodified EF-P leads to a reduction in the translation efficiency of polyproline-containing proteins [19]. *Pseudomonas aeruginosa* EF-P is rhamnosylated by the EarP enzyme, which significantly increases antibiotic resistance [8]. *Bacillus subtilis* EF-P 5-aminopentanolylation by the YmfI protein is required for swarming motility [20]. These different modifications of EF-P modulate bacterial phenotypes. STK, an important phosphokinase, has been reported to play multiple roles in *S. suis* pathogenesis [9, 10, 13, 21, 22]. To date, several substrates of STK have been shown to modulate cell division, peptidoglycan synthesis, capsular synthesis, oxidative stress resistance and virulence. Our study revealed for the first time that STK phosphorylates EF-P at T176. Owing to the difficulty in *efp* deletion in SC19 and the importance of EF-P modification in its function, phosphorylated and nonphosphorylated EF-P states were mimicked by constructing SC19 mutants with chromosomally integrated pSET2-EF-P or pSET2-EF-P-T176A, respectively, to investigate the effect of *S. suis* EF-P phosphorylation on bacterial virulence. Interestingly, the phosphorylation of EF-P at S148 by STK was also observed. Unfortunately, due to the difficulty in generating S148 mutants, we focused on the effect of EF-P phosphorylation at T176 on BBB disruption. However, the functional significance of S148 phosphorylation needs to be further studied. Our study revealed that EF-P phosphorylation at T176 facilitated *S. suis*-induced BBB disruption. The lack of reports on EF-P phosphorylation in other bacterial species suggests a specific adaptation in *S. suis*. This exclusive modification in *S. suis* indicates the evolutionary pressure to fine-tune translation under host stress conditions. Phosphorylation enables EF-P activation in response to host defence during infection, which is similar to eukaryotic eIF5A, whose hypusination is dynamically regulated during stress. However, whether EF-P phosphorylation is conserved in other bacterial species needs to be further studied.

The discovery that EF-P mediates the synthesis of proteins containing consecutive polyproline motifs provides a unifying mechanism for the physiological consequences triggered by the loss of EF-P. To date, few proteins are affected by the loss of EF-P. A lack of EF-P increases bacterial sensitivity in response to stress conditions. EF-P mediates the expression of the polyproline-containing pH receptor CadC in response to acid stress [7]. In *Salmonella*, EF-P is required for translation of the *mgtB* gene encoding a Mg^2+^ transporter, but substitution of P555 and P556 with alanine abolishes EF-P-mediated *mgtB* translation and thereby promotes *Salmonella* survival in macrophages and virulence in mice [23]. Our study revealed for the first time that EF-P in *S. suis* mediates the expression of SP containing three consecutive proline motifs at P178-P180. In addition, recombinant SP led to a reduction in the expression of the TJ protein ZO-1 and TEER value in human brain endothelial cells (hCMEC/D3) to disrupt the BBB, which is consistent with the ability of SP to cleave proteins in other bacterial species. SP deficiency alleviated bacterium-induced BBB disruption, whereas the complementation mutant CΔ*B9H01_03990* abolished the reduction in BBB disruption, indicating that SP plays an important role in destroying the host barrier. The SP HtrA in *Campylobacter jejuni* cleaves TJ caudin-8 to destroy the intestinal barrier [24]. The SP in *Neisseria meningitidis* exhibits cleavage activity against IgA and IgG to promote its invasion [25]. Although EF-P has been determined to target polyproline motifs, the factors that drive EF-P-mediated translation are complex. EF-P has also been reported to target specific regions without polyproline motifs in *Salmonella enterica* and *Escherichia coli* [26]*.* The mutation of any residue within a specific region abolishes the requirement of EF-P. In our study, although we did not directly identify the specific region in which EF-P targets SP, we found that the increase in EF-P activity upregulated EF-P expression, indicating that EF-P mediates SP translation. However, the specific sequence that EF-P targets and whether the target region affects EF-P function need to be further studied.

In conclusion, we revealed that EF-P is a novel substrate of STK and that EF-P phosphorylation is required for *S. suis*-induced BBB disruption. In addition, we revealed that EF-P phosphorylation mediates SP expression, thereby promoting *S. suis*-induced BBB disruption. These findings elucidate a novel interplay between STK, EF-P and SP in the regulation of *S. suis*-induced BBB disruption, which provides new insight into the pathogenesis of *S. suis*.

## Supplementary Information

**Additional file** **1. EF-P Mass spectra.** A mass spectra showing EF-P phosphorylation at Thr-12, Ser-26, Thr-35, Thr-144, Ser-148, Thr-176, Thr-180, Ser-183.

**Additional file** **2. Primer information used in this study**.

## Data Availability

Not applicable.
