# Supplementary figures and images for: Phosphorylation of EF-P aggravates Streptococcus suis-induced blood–brain barrier damage by enhancing serine protease production
Source: Vet Res. 2025 Nov 7;56:215. doi: 10.1186/s13567-025-01612-x (PMC12595652; doi:10.1186/s13567-025-01612-x)

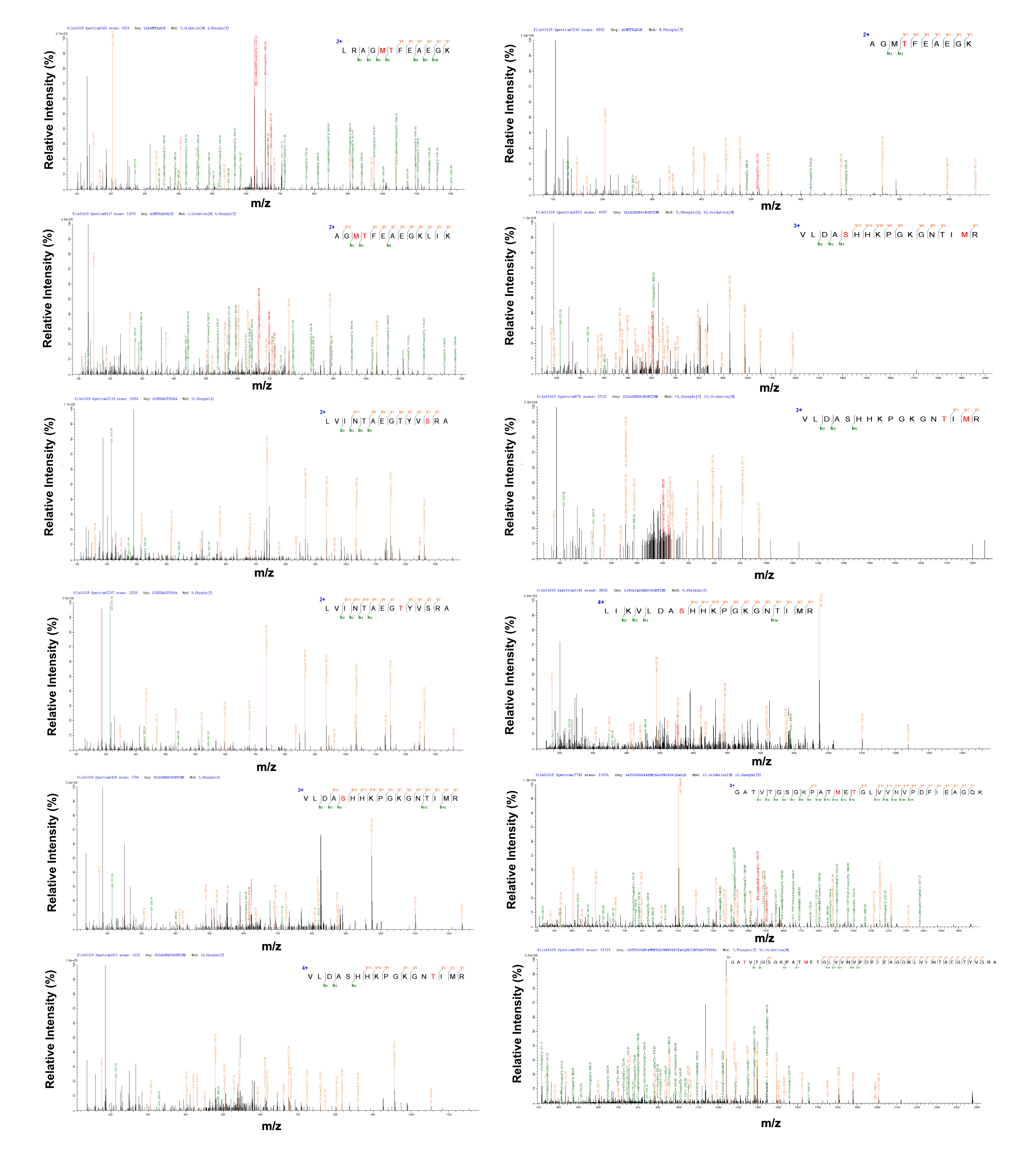

Supplement: Supplementary file 1 — Additional file 1. EF-P Mass spectra. A mass spectra showing EF-P phosphorylation at Thr-12, Ser-26, Thr-35, Thr-144, Ser-148, Thr-176, Thr-180, Ser-183. [file 13567_2025_1612_MOESM1_ESM.tif]
